# Distinct polyamine effects on *Candida albicans* morphogenesis identify spermidine as a key regulator

**DOI:** 10.1128/spectrum.00833-26

**Published:** 2026-09-23

**Authors:** Ruvini U. Pathirana, Minelly I. Camargo, Geysira G. Vela, David Kadosh

**Affiliations:** 1 Department of Biology & Chemistry, Texas A & M International University 4420 , Laredo, Texas, USA; 2 Department of Microbiology, Immunology, and Molecular Genetics, University of Texas Health Science Center at San Antonio 14742, San Antonio, Texas, USA; Debreceni Egyetem, Debrecen, Hungary

**Keywords:** *Candida albicans*, polyamines, filamentation, spermine, ornithine decarboxylase, spermidine

## Abstract

Polyamines play essential and multifaceted roles in all organisms. However, their unique functions in fungal cell differentiation provide an opportunity to identify novel antifungal targets, as inhibition of essential metabolic pathways represents a promising therapeutic strategy. In this study, we characterized two putative regulators of the polyamine biosynthesis pathway in the major human fungal pathogen *Candida albicans*, identified based on their homology to *Saccharomyces cerevisiae SPE1* and *SPE2*, which encode ornithine decarboxylase and *S*-adenosylmethionine decarboxylase, respectively. We generated single and double gene deletion strains for Ca*SPE1* and Ca*SPE2* and found that disruption of these genes severely impaired yeast cell growth due to disturbed polyamine homeostasis. This dysregulation was associated with pronounced cell vacuolar enlargement, indicating that cellular stress is linked to polyamine imbalance. Filamentation analyses revealed that polyamine-induced morphogenesis by spermine and spermidine is growth medium-dependent, likely reflecting differences in the bioavailability of different types of polyamines. Among the polyamines tested, spermine acted synergistically with glucose to promote filamentation in minimal medium. Collectively, our findings demonstrate that polyamines mediate a finely tuned balance between yeast cell proliferation and filamentous growth in *C. albicans*. Notably, spermidine directly regulates polyamine homeostasis to govern cell growth and filamentation, and spermine appears to be the primary polyamine driving filamentation, potentially through a dual signaling mechanism that integrates metabolic status and morphogenetic regulation.

**IMPORTANCE:**

Metabolic plasticity is a critical determinant of microbial commensalism and pathogenicity, shaped by the evolutionary pressure that drives niche-specific adaptations. These processes are not well understood in a species-specific context across diverse morphogenetic programs that govern fungal adaptations. As a major dimorphic human fungal pathogen, *Candida albicans* finely tunes its metabolic pathways to support its growth and virulence properties, including morphogenesis. Here, we investigated one such understudied pathway: polyamine metabolism under distinct growth conditions. Building upon previous studies on fungal polyamine biology, we demonstrated that polyamine homeostasis influences growth, cell morphology, and filamentation in *C. albicans*. This study expands our understanding of how diverse polyamines shape *C. albicans* physiology by providing insight into the dual roles of polyamines as nutrients and signaling molecules. These findings illustrate the complex relationship between polyamine metabolism and morphogenesis in *C. albicans* while identifying polyamine metabolism as a pathway of continued interest for antifungal target discovery.

## INTRODUCTION

Our understanding of the diverse metabolic pathways in the human opportunistic fungal pathogen *Candida albicans* is expanding, shedding light on the fine distinctions between its commensal lifestyle as a dominant member of the human microbiome and its opportunistic, potentially life-threatening, pathogenic lifestyle in immunocompromised patients. Significant research on the pathobiology of *C. albicans* over the last few decades (1) also highlights the importance of studying *C. albicans* not only as a pathogen but also as a model organism to improve our understanding of fungal physiology and morphology in order to identify novel drug targets (2). Nutrient assimilation and adaptive strategies play a vital role in the evolution and establishment of fungal-host relationships across diverse habitats and exhibit remarkable metabolic flexibility (2, 3). While some metabolic pathways are highly conserved, others have undergone significant diversification, resulting in unique biosynthesis pathways or metabolic products. Such metabolites support not only cell growth and differentiation but also act as mediators of virulence-related traits such as morphogenesis, quorum sensing, and stress tolerance (2).

**Fig 1:**
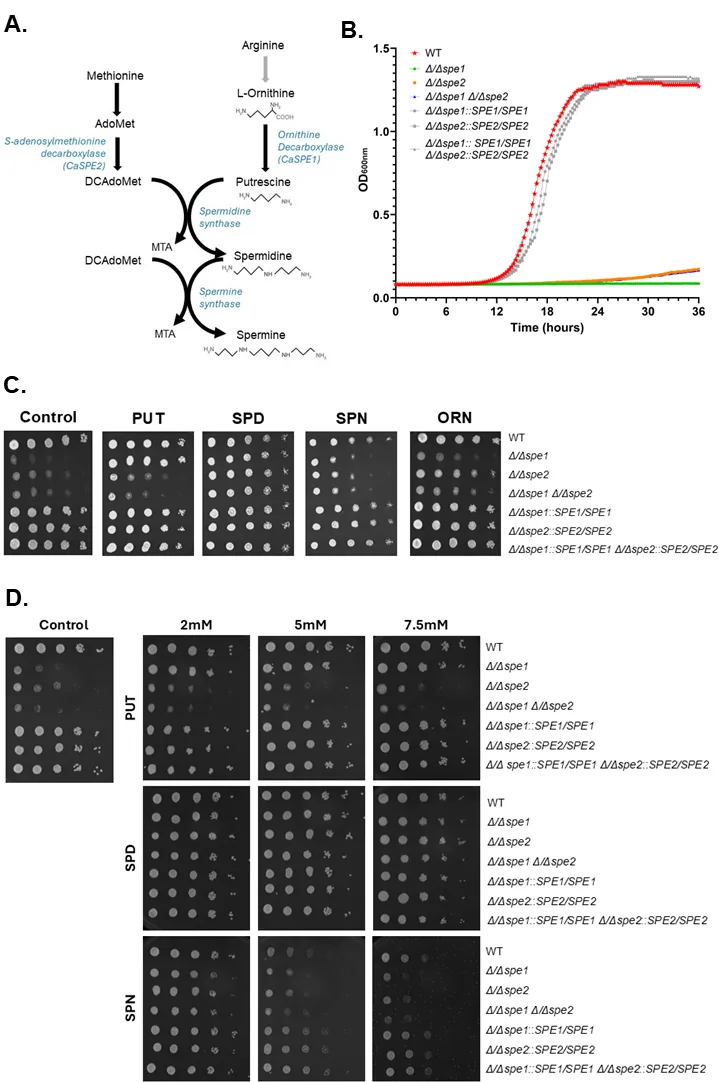
*SPE1* and/or *SPE2* deletion drastically reduces *C. albicans* growth. (**A**) Putative polyamine biosynthesis pathway in yeasts. In *C. albicans* (*Ca*), *SPE1* encodes for ornithine decarboxylase (ODC) activity and *SPE2* encodes for *S-*adenosylmethionine decarboxylase (AdoMetDC) activity. (**B**) Growth curves of polyamine-deficient mutants compared to those of WT and complemented strains, as measured by OD_600nm_ during growth in polyamine-free SC liquid medium. (**C**) Spot assays showing rescue of polyamine-deficient mutant growth defects with added polyamines, putrescine (PUT), spermidine (SPD), spermine (SPN), and the starting substrate, ornithine (ORN). (**D**) Growth defect rescue phenotypes of polyamine-deficient mutants under varying concentrations (2, 5, and 7.5 mM) of putrescine (PUT), spermidine (SPD), and spermine (SPN).

Among different metabolites studied in detail in *C. albicans*, one of the overlooked groups of molecules is polyamines. Although the role of polyamines in *C. albicans* filamentation is well known (4–9), their specific roles in *C. albicans* physiology have not yet been determined. Polyamines are polycations that contain more than one amino group and are ubiquitous in nature due to their prominent essential roles in all biological systems. The most common polyamines include the diamine putrescine, the triamine spermidine, and the tetraamine spermine. Due to their positive charge, polyamines can interact with various negatively charged cellular components such as DNA, RNA, and phospholipids (10–12). Consequently, polyamines are important regulators of gene expression (via chromatin remodeling), cell growth and proliferation, cell lifespan, and cellular stress responses (10–16). Excessive polyamines inside cells are known to cause deleterious effects, likely by binding indiscriminately to biomolecules and producing reactive metabolic byproducts, which lead to cell damage and death (17–19). Across all kingdoms of life, intracellular polyamine levels are tightly regulated by fine-tuning of their biosynthesis, catabolism, and transport. Interestingly, putrescine is the major polyamine in *Escherichia coli*, while spermine and spermidine are prominent in many other bacteria and eukaryotes (20). This variation is most likely due to species-specific biosynthesis pathways and the presence of diverse regulatory mechanisms to avoid abrupt polyamine depletion and accumulation (14, 19, 21). So far, the highly conserved ornithine decarboxylase (ODC) enzyme has been identified as the rate-limiting step for polyamine biosynthesis in most organisms, including *Saccharomyces cerevisiae* and other fungi such as *Neurospora crassa* and *Schizosaccharomyces pombe* (22–25). The canonical pathway for *de novo* polyamine biosynthesis (Fig. 1A) starts with decarboxylation of ornithine by ODC to generate putrescine and proceeds further with the sequential addition of two aminopropyl groups via methionine-derived decarboxylated *S*-adenosylmethionine (DCAdoMet) to produce spermidine and spermine, respectively. The latter reaction is catalyzed by *S*-adenosylmethionine decarboxylase (AdoMetDC) enzyme activity. However, arginine catabolism is the main source for polyamine biosynthesis for most cells and occurs via arginine decarboxylase (ADC) activity (19, 26). Apart from arginine, agmatine and/or lysine can be used by certain organisms as secondary sources for polyamine biosynthesis (15, 19). Interestingly, ADC is limited to higher eukaryotes, while ODC serves as the main or sole producer of polyamines via ornithine in lower eukaryotes such as fungi (16).

The presence of the ODC gene in *C. albicans*, which has significant homology to those of *S. cerevisiae* and *N. crassa* (5), was first confirmed through inverse PCR technique by Lopez et al. (1997). This gene has been annotated as *SPE1* (27) and encodes a protein of 473 amino acids, which is important for growth and filamentation (4, 9). *C. albicans* also possesses a *SPE2* gene homolog, which encodes a putative AdoMetDC of 381 amino acids in length. *SPE1* is essential for invasive growth and full virulence in a murine model of oral candidiasis (7). In the presence of 1,4-diamino-2-butanone (DAB), a competitive inhibitor of ODC, *C. albicans* showed a sharp decrease in polyamine content accompanied by reduced adenylyl cyclase (Cyr1) activity and reduced cAMP content, suggesting that polyamines function upstream of the cAMP-PKA signaling pathway (9). Interestingly, decarboxylated *S*-adenosyl methionine (SAM, resulting from methionine), a co-substrate for ornithine-mediated polyamine biosynthesis, has been found to be the regulatory molecule for methionine-induced *C. albicans* filamentation mediated by the cAMP-PKA pathway (8). Furthermore, this finding linked coregulatory functions of methionine and a polyamine as a target for filamentation control. Strikingly, Schrevens et al. (8) also found that *SPE2* deletion leads not only to impaired filamentation but also defective biofilm formation and reduced virulence in a disseminated *C. albicans* infection model (8). Finally, in two separate DNA microarray studies, upregulation of *SPE2* expression (28) and downregulation of *SPE1* (29) were observed during the early phase of *C. albicans* biofilm formation (adherence).

Although previous studies have confirmed the important roles of polyamines in the morphogenesis of *C. albicans* (4, 9), our knowledge of polyamine metabolism and its regulation is fragmentary and incomplete for this dimorphic fungal pathogen. Therefore, in this study, we will gain a better understanding of the interdependent roles that *SPE1* and *SPE2* play in *C. albicans* polyamine biosynthesis, growth, and morphogenesis. The canonical pathway for polyamine biosynthesis converges just downstream from the *SPE1* and *SPE2* catalytic steps (Fig. 1A). Therefore, we constructed single and double gene deletion mutants for *SPE1* and *SPE2* (30). We hypothesized that in the absence of *SPE1* and/or *SPE2*, *C. albicans* would be unlikely to grow if these enzymes represent the sole route for polyamine biosynthesis. Here, we report that deletion of neither *SPE1* nor *SPE2* completely abolishes the polyamine pool but rather dramatically disrupts polyamine homeostasis. The resulting accumulation of various polyamine types and/or toxic byproducts is likely the major cause of observed abnormal cell growth and eventually cell death. Interestingly, we found that spermine is the major polyamine responsible for filamentation in *C. albicans* through a synergistic interaction with glucose. Our results further suggest that polyamines play a dual role in cell signaling to regulate both *C. albicans* cell growth and morphogenesis.

## RESULTS

### Both *SPE1* and *SPE2* are essential for normal growth of *C. albicans*

To identify the possible routes of polyamine biosynthesis in *C. albicans*, we first blocked the putative polyamine biosynthesis pathway through the deletion of two genes, namely *SPE1* (C1_00740C/orf19.6032), with putative ODC activity, and *SPE2* (C5_00700C/orf19.568), with putative *S*-adenosylmethionine decarboxylase activity in the SC5314 wild-type (WT) strain background. Since the enzymatic activities of these genes have not yet been annotated, we verified these genes exclusively based on sequence homology (Fig. S1). The respective homologs from the related yeasts *S. cerevisiae* and *S. pombe* have been well characterized and are likely to share the same protein functions due to their high similarity indices (Fig. S1).

We first assessed the growth kinetics of isogenic mutant strains of *Δ/Δspe1*, *Δ/Δspe2,* and *Δ/Δspe1 Δ/Δspe2* in polyamine-free synthetic complete (SC) medium. We observed that the growth rate was significantly reduced in all mutants in the absence of polyamines and that these growth defects were reversed by the add-back of the deleted gene (Fig. 1B and C). None of the mutants were able to grow optimally as the wild-type strain upon subculturing into the fresh liquid/solid SC minimal medium (data not shown), indicating that the slight growth observed in the initial culture likely resulted from residual intracellular polyamine stores. Therefore, both *SPE1* and *SPE2* function as conditional essential genes that are required for normal cell growth and proliferation under conditions of limited polyamine availability.

Next, we examined how the exogenous addition of polyamines or substrate for polyamine biosynthesis could rescue these growth defects through spot assays on polyamine-free SC media (Fig. 1C). Spermidine fully rescued the growth of all mutants. Although putrescine completely rescued the *Δ/Δspe1* growth defect, both *Δ/Δspe2* and *Δ/Δspe1 Δ/Δspe2* mutants were unable to grow optimally in the presence of putrescine likely because *SPE2* deletion causes a halt to the smooth carbon flow of the pathway from putrescine onward. Although spermine alone supported the growth of all mutants to some extent, none of the mutants were able to achieve optimal growth, possibly due to the lack of upstream polyamines, spermidine, and/or putrescine. Ornithine alone supported the growth of the *Δ/Δspe2* mutant, but not *Δ/Δspe1* or *Δ/Δspe1 Δ/Δspe2* mutants, emphasizing the importance of *SPE1* for the conversion of ornithine to putrescine. Collectively, these observations indicate that while polyamines are important for optimal growth of *C. albicans*, the utilization of specific polyamine types (putrescine, spermidine, or spermine) is dependent on pathway integrity and mutant background, with spermidine serving as the key polyamine sufficient to restore optimal growth in all mutants.

### Dose-dependent effect of polyamines on *C. albicans* growth

Although polyamines are essential components of all living cells, excessive polyamine levels have been shown to have detrimental effects. Due to this dose-dependent bi-lateral role of polyamines, we investigated the possible toxic effects of polyamine imbalance by adding excess polyamines. More specifically, we grew WT and mutant strains in SC medium with added polyamines (putrescine, spermidine, and/or spermine) in a range of concentrations (Fig. 1D). Interestingly, all the mutant growth phenotypes were rescued by adding low levels of spermidine and spermine (2 mM). Higher concentrations of spermine (5 mM and 7.5 mM) retard the growth of all strains, including the WT strain. All mutant strains showed increased tolerance to higher concentrations of spermidine (7.5 mM) but not spermine. In contrast, all concentrations of putrescine did rescue the growth defect of the *Δ/Δspe1* mutant but not that of *Δ/Δspe2* or *Δ/Δspe1 Δ/Δspe2* mutants. The growth recovery by each polyamine depended on the gene deleted. Overall, our results indicate that certain mutants showed differential sensitivities to elevated polyamine concentrations, consistent with the direction of the carbon flux in the canonical pathway for polyamine biosynthesis (Fig. 1A), further underscoring the importance of polyamine homeostasis for normal cellular growth.

### Deletion of *SPE1* and *SPE2* alters intracellular polyamine homeostasis

**Fig 2:**
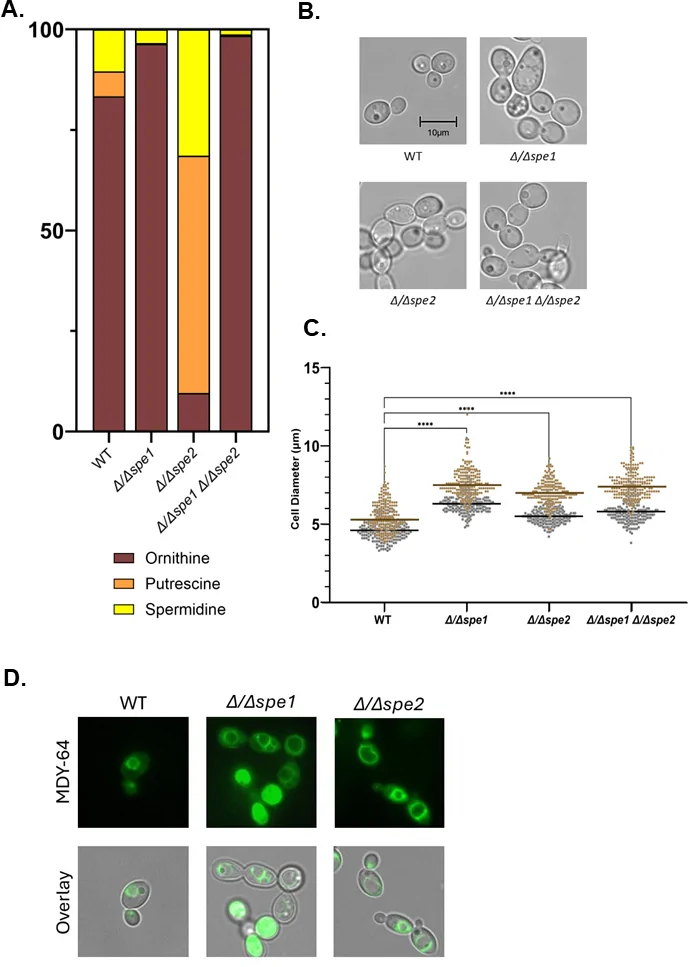
Altered polyamine homeostasis leads to abnormal *C. albicans* cell morphology. (**A**) Polyamine content is shown for each indicated mutant strain compared to that of the WT strain. The stacked bar graph shows the percentage content of polyamines (Y-axis in %). The average of at least three biological replicates was used for normalization via fraction of total analysis on GraphPad Prism 10 software. The averages of at least three biological replicates for each sample were used for normalization via fractions of total and presented as a stacked bar graph, where 100% is the sum of all means in the data set. (**B**) Representative images of cell morphology for each indicated strain. (**C**) Measurement of cell dimensions (µm) after 24 h of growth in minimal medium at 30°C showing that mutant strains with abnormal cell shapes are significantly larger when compared to the WT strain. The cell width (W) is indicated in gray, and length (L) is indicated in golden yellow. Each dot corresponds to one yeast cell. The horizontal bar indicates the median of each data set in respective color. (**D**) Representative fluorescent images of the indicated strains, WT, *Δ/Δspe1,* and *Δ/Δspe2*, following vacuolar staining with MDY-64 (Invitrogen). Yeast cells grown to stationary phase in minimal media were stained with MDY-64 and observed by fluorescence microscopy.

Next, using liquid chromatography–mass spectrometry (LC-MS), we analyzed the polyamine content in *SPE1-* and/or *SPE2*-deleted mutants using cell pellets harvested from growth in polyamine-free medium. Relatively, ornithine content is high in the WT strain compared to other polyamines in the pool (Fig. 2A). This result suggests the regulated flow of ornithine toward polyamine biosynthesis or possibly even other cellular processes. In the absence of *SPE1*, ornithine accumulated in both *Δ/Δspe1* and *Δ/Δspe1 Δ/Δspe2* mutants by approximately 0.8-fold compared to that in the WT strain. Similarly, putrescine content is significantly reduced in both *Δ/Δspe1* and *Δ/Δspe1 Δ/Δspe2* mutants by more than approximately 26-fold and 19-fold, respectively. Reduction in putrescine and subsequent intermediates in the pathway was expected in the absence of ODC activity according to the canonical polyamine biosynthesis pathway in yeast (Fig. 1A). Deletion of *SPE2* greatly reduced ornithine content by 8.7-fold but remarkably increased putrescine (10-fold) and spermidine (3-fold) content, in agreement with reduced carbon influx to the downstream pathway in the absence of DCAdoMet decarboxylation (i.e., *SPE2* activity). The putrescine content was markedly reduced in *Δ/Δspe1* and *Δ/Δspe1 Δ/Δspe2* mutants, likely due to the interrupted *SPE1* activity, which results in ornithine accumulation. Deletion of *SPE1* abolished putrescine production, while deletion of *SPE2* causes putrescine accumulation. Interestingly, the double mutant lacking both *SPE1* and *SPE2* showed very little putrescine. This pattern suggests that *SPE1* and *SPE2* function through a coregulatory mechanism rather than through independent cumulative effects. Our assay did not detect spermine in any of the strains. This finding is not surprising since in previous studies none of the assay methods detected any spermine in fungi (31, 32).

### Deletion of *SPE1* and *SPE2* results in abnormal cell shapes

Despite the very slow growth rates, we observed distorted cell morphologies for *C. albicans Δ/Δspe1*, *Δ/Δspe2,* and *Δ/Δspe1 Δ/Δspe2* mutants. We quantified the different cell sizes by measuring the length (longest diameter) and the width (shortest diameter) of at least 200 cells from each strain (Fig. 2B and C). Compared to the WT strain, the mutant cells are significantly larger in both length and width (Mann-Whitney test, *P* < 0.0001). The mutant cell sizes were also distributed on a wider scale than that of the WT strain. We also observed by staining with MDY-64 that both *Δ/Δspe1* and *Δ/Δspe2* mutant cells have abnormally enlarged vacuoles (Fig. 2D; Fig. S2). Vacuole staining revealed typical single, large vacuoles in wild-type yeast cells, appearing as distinct structures, while all mutant yeast cells showed either enlarged vacuoles, or multiple vacuoles (fragmentation) in certain cases, which are characteristic of cell stress conditions. Closer inspection of the vacuolar membrane showed irregularities or invaginations in a portion of mutant cells, which likely depends on the amount of osmotic pressure that occurs or cell population heterogeneity due to polyamine imbalance.

### Both *SPE1* and *SPE2* are required for *C. albicans* filamentation

Polyamines are known to induce filamentation of *C. albicans* (4, 9). In agreement with previous observations, we showed that spermine, spermidine, and putrescine can induce filamentation in a WT *C. albicans* strain grown in YPD liquid medium (Fig. S3A). This filament-inducing ability is observed at lower polyamine levels (≤10 mM), while at higher polyamine levels (20 mM), cells grow in the yeast form (Fig. S3B), likely due to the toxic effects of polyamines at higher concentrations.

**Fig 3:**
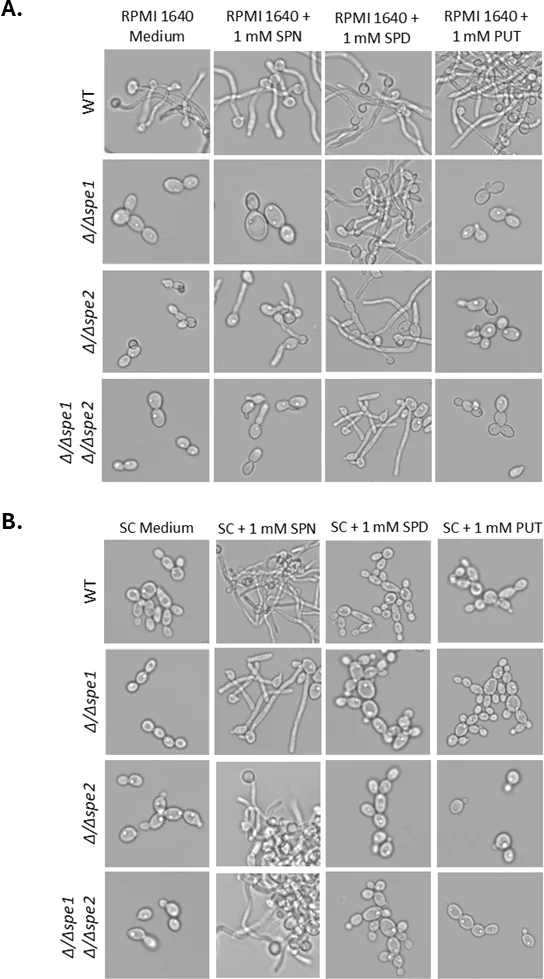
Polyamines drive *C. albicans* filamentation, showing medium-dependent variation in hyphal growth. (**A**) Representative bright-field images of cell morphology following hyphal induction in RPMI 1640 medium supplemented with respective polyamines at 37°C for 4 h. Exogenously added spermidine restored filamentation of polyamine-deficient mutants. Further, *SPE1* deletion (*Δ/Δspe1* mutants) locked *C. albicans* in yeast form, while *Δ/Δspe2* and *Δ/Δspe1 Δ/Δspe2* mutants weakly induced germ tubes in RPMI 1640 medium in the presence of exogenous spermine. (**B**) Representative bright-field images of cell morphology following hyphal induction in SC + 0.2% glucose medium with respective polyamines at 37°C for 4 h. Added spermine, but not spermidine nor putrescine, fully restored filamentation of polyamine-deficient mutant phenotypes in SC + 0.2% glucose medium. SC, Synthetic complete medium; PUT, putrescine; SPD, spermidine; SPN, spermine.

We then tested the filamentation ability of *C. albicans Δ/Δspe1*, *Δ/Δspe2*, and *Δ/Δspe1 Δ/Δspe2* mutants. For inoculum preparation, cells grown on polyamine-free SC agar plates were inoculated in polyamine-free SC liquid medium (with 2% glucose) and grown for 28–30 h before harvesting. This ensured that the cells either used *de novo* synthesized polyamines (in the WT strain) or leftover internal polyamine reserves. We observed that our polyamine-deficient mutants indeed did not form filaments in RPMI 1640 medium (Fig. 3A). To verify the phenotype, RPMI 1640 medium was supplemented with different polyamines. Interestingly, spermidine, but not spermine or putrescine, fully restored filamentation of all mutants (Fig. 3A). This finding shows that not all polyamine types are equally capable of inducing filamentation in nutrient-limited media such as RPMI 1640, suggesting that not all polyamines may act as direct filament inducers. Instead, this result is likely to reflect the importance of polyamine homeostasis, rather than the mere presence of individual polyamines, for filamentation. It was also noted that *Δ/Δspe2* and *Δ/Δspe1 Δ/Δspe2*, but not *Δ/Δspe1*, mutants, in certain instances attempted to form germ tubes in the presence of polyamines, but they never fully elongated. This observation could be attributed to any remaining internal polyamines signaling filamentation or the presence of a synergistic effect with another non-polyamine signaling molecule. Additionally, as expected, all add-back strains (*Δ/Δspe1::SPE1/SPE1*, *Δ/Δspe2::SPE2/SPE2,* and *Δ/Δspe1::SPE1/SPE1 Δ/Δspe2::SPE2/SPE2*) showed restored filamentation in RPMI 1640 medium (Fig. S4).

To further elucidate the additional requirements for polyamine-induced filamentation, we repeated these experiments using minimal medium (SC liquid medium) instead of RPMI 1640. We first assessed the ability of the WT strain to undergo filamentation in SC medium with 2% glucose and in the presence of spermine, spermidine, or putrescine. The glucose concentration was then adjusted to 0.2% to match that of RPMI 1640 media. Since filamentation was robust under both 0.2% and 2% glucose levels, we continued subsequent filamentation experiments in SC medium with 0.2% glucose to maintain consistency with RPMI 1640 conditions. The WT strain showed robust filamentation in SC medium supplemented with spermine, as in the case of growth in RPMI 1640. However, no significant change in filamentation was observed in the presence of spermidine or putrescine (Fig. 3B). Notably, all the polyamine biosynthesis-deficient mutant strains also showed restored filamentation in the presence of spermine but not spermidine or putrescine. Collectively, these findings indicate that spermine specifically promotes filamentation under minimal nutrient conditions, reinforcing the idea that distinct polyamine types differentially regulate morphogenesis and that spermine (directly or generated through spermidine), possibly in concert with glucose, plays a unique and non-redundant role in this process.

### Glucose is essential for the spermine-induced filamentation in *C. albicans*

**Fig 4:**
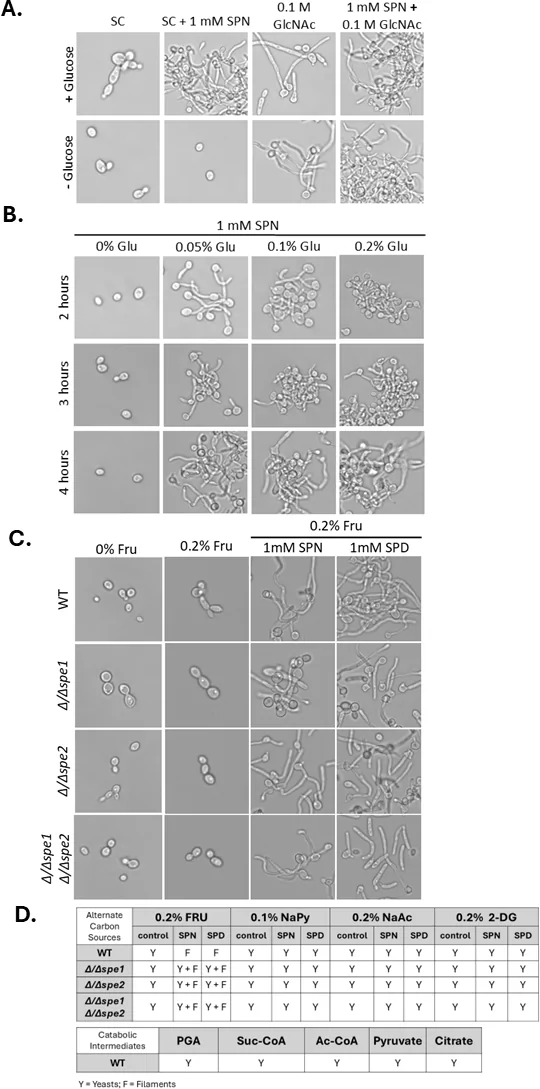
Spermine acts as the major polyamine functioning synergistically with glucose to induce *C. albicans* filamentation. (**A**) Spermine (SPN) induced filamentation in the WT strain only in the presence of glucose (Glu). (**B**) Time-lapse images of filament formation in the WT *C. albicans* strain in the presence and absence of 0%, 0.05%, 0.1%, and 0.2% glucose. Spermine-induced filamentation requires the presence of glucose, even at low concentrations. (**C**) Fructose (Fru) can replace glucose requirement for polyamine-induced filamentation in *C. albicans*. Representative bright-field images of cell morphology following hyphal induction in SC + 0.2% fructose with respective polyamines, spermine (SPN) or spermidine (SPD) at 37°C for 4 h. (**D**) Alternate carbon sources, except fructose, or glycolytic or Krebs cycle intermediates, were unable to restore filamentation in place of glucose in spermine-containing minimal medium. Alternate carbon sources used include 0.2% FRU: fructose, 0.1% NaPy: sodium pyruvate, 0.2% NaAc: sodium acetate, and 0.2% 2-deoxyglucose (2-DG). Glycolytic and Krebs cycle intermediates include 0.2% phosphoglyceric acid (PGA), 0.2% succinyl-Coenzyme A (Suc-CoA), 0.2% acetyl Coenzyme A (Ac-CoA), 0.2% pyruvate, or 0.2% citrate.

Based on our previous observations (Fig. 3), we hypothesized that spermine acts synergistically with glucose to induce filamentation in *C. albicans*. To test this hypothesis, we examined the requirement of glucose for spermine-induced filamentation of a WT strain by assessing morphology in SC medium in the presence vs absence of glucose as the carbon source. The WT strain did not form any filaments in the absence of glucose in SC medium supplemented with spermine (Fig. 4A). We extended our experiments by titrating glucose concentration and found that even a concentration of 0.05% glucose is sufficient to induce filamentation when spermine is present (Fig. 4B). This finding confirmed that spermine-induced filamentation occurs only in the presence of added glucose to the minimal medium.

Since glucose can act as both a nutritional and signaling molecule in *C. albicans* (33), we next wanted to determine whether glucose functions as a synergistic partner in this spermine-induced filamentation. We replaced glucose with different carbon sources (glycolytic and Krebs cycle pathway intermediates) in the presence of either spermine or spermidine as the main polyamine counter partner. The substitute carbon source molecules included fructose, sodium pyruvate, sodium acetate, and 2-deoxyglucose (2-DG). Glucose-6-phosphate (G-6-P) is the first phosphorylated intermediate in glycolysis and serves as a key metabolic branch point that can direct glucose toward catabolism. Additionally, other key intermediates, such as fructose 1,6-bisphosphates, can induce filamentation in *C. albicans* (34). These substitutions allowed us to dissect the specific metabolic requirements as well as direct and indirect effects of glucose for polyamine-induced filamentation in *C. albicans*. The inability of a WT strain or polyamine biosynthesis-deficient mutants to induce filaments in the absence of glucose (Fig. 4) confirmed that glucose is likely a synergistic signaling molecule for spermine. Except for fructose, none of the alternate carbon sources or carbon catabolites were able to induce filamentation in the presence of spermine or spermidine (Fig. 4C and D). Fructose is a monosaccharide comparable to glucose and has been reported to induce filamentation in *C. albicans* (34, 35). Furthermore, the WT strain was unable to induce filamentation in the presence of the glycolytic intermediates phosphoglyceric acid (PGA) and the Krebs cycle intermediates succinyl-Coenzyme A (Suc-CoA), acetyl Coenzyme A (Ac-CoA), or citrate. However, we acknowledge that the lack of filamentation observed, particularly with Co-A derivatives, should be interpreted with caution, as these relatively large molecules may not be readily taken up by yeast cells. We also used 2-DG, a non-metabolizable glucose analog, as a control to differentiate the effects of glucose sensing from those associated with glucose metabolism. Consistent with its inability to undergo glycolysis, 2-DG did not promote filamentation (Fig. 4D), indicating that glucose sensing alone is insufficient and that 2-DG does not increase intracellular glycolytic intermediates. Together, these findings suggest that glucose or glucose-derived sugar phosphates serve as the synergistic signaling molecule(s) for spermine-induced filamentation in *C. albicans*.

## DISCUSSION

Polyamines are nutritionally essential to humans and all other biological organisms at very small concentrations, yet they have toxic effects at higher concentrations. Polyamines have also been shown to exert pleiotropic effects on fungal growth and fungal morphogenesis, such as dimorphism in *C. albicans* (4, 5, 9), *Paracoccidioides brasiliensis* (36, 37), *Yarrowia lipolytica* (38–40), and *Ustilago maydis* (41, 42); conidiation in filamentous fungi such as *Aspergillus nidulans* (43, 44); and appressorium formation in *Magnaporthe grisea* (45, 46). However, mechanisms that drive polyamine-induced filamentation in fungi are not well characterized. In this study, we explored the polyamine-dependent trade-off between growth and filamentation in *C. albicans* in relation to the polyamine biosynthetic pathway. The ornithine-dependent polyamine biosynthesis pathway (Fig. 1A) has been shown to be conserved among at least all yeasts (14, 47), including *C. albicans* (this study). The defective growth phenotypes, based on growth rate and abnormal cell morphologies, in polyamine-deficient *C. albicans* mutant strains used in this study confirmed that *SPE1* is the rate-limiting step for *C. albicans* polyamine biosynthesis. The remnant growth of *C. albicans* indicated the possible presence of stored polyamines, which were identified through LC-MS analysis. We presume that the depletion of intracellularly stored polyamines is slow. However, due to the absence of polyamine homeostasis, cells likely encounter stress conditions before the internal stores are completely depleted. For our assays, we grew all our cells in polyamine-free minimal medium to deplete polyamine reserves as much as possible. Mutant cells pre-grown in YPD continued to grow for at least 2–3 generations in minimal medium, although more slowly than those of the WT strain. Following serial passage in polyamine-free media, we did not observe any evidence of sustained growth recovery; instead, the growth progressively declined, indicating that successive passages are needed to deplete residual intracellular polyamines and fully reveal the mutant phenotype. Further, the polyamine defective growth phenotypes of *C. albicans* identified in this study resemble those of *S. cerevisiae* and *Schizosaccharomyces pombe* strains deleted for *SPE1* and *SPE2* observed by Tabor et al. (22, 25, 48, 49), indicating that polyamine biosynthesis mutants can undergo a limited number of cell divisions supported by pre-existing intracellular polyamine pools before growth ceases. Our results further verify that ornithine-induced polyamine biosynthesis is the sole pathway in *C. albicans* as in *S. cerevisiae*. We cannot exclude the possibility of putative substrates for polyamine or its derivatives, such as agmatine, that could contribute to the overall metabolic pool in *C. albicans* (50). However, we believe that their influx may not be sufficient to satisfy the cell polyamine demand to maintain homeostasis in the absence of *SPE1* or *SPE2* function. The analysis of polyamine content (Fig. 2A) in each mutant strain compared to that of the WT strain showed that polyamine flux correlates with the *SPE1*-mediated canonical pathway in yeasts. Interestingly, in agreement with previous studies, we also did not detect spermine (31, 51). This might be due to either very low concentrations of spermine, which are not detectable by our analysis method, or the rapid conversion of spermine into subsequent end products. However, a possible retroconversion mechanism, where spermine is converted into putrescine through acetylation or protected from reversion through deacetylation to prevent its transformation into putrescine, could also account for this observation (52, 53).

The abnormal vacuole shapes observed in polyamine biosynthesis-deficient mutant cells may be due to the cells attempting to reverse unwanted changes in ion content. The enlargement of vacuoles is strongly associated with higher polyamine transporter activity in the vacuole membrane (54). The enlarged vacuoles observed in our polyamine-deficient mutants suggest that these cells are overexpressing the polyamine transporters, and possibly other transporters, to regulate excessive ion accumulation from positively charged polyamines and prevent cell toxicity. Yeast vacuoles can undergo two morphogenetic changes, fragmentation and degradation, in response to cell stress and autophagy, respectively (55). Vacuolar irregularities we observed in polyamine-deficient mutants in this study closely resemble fragmentation, rather than degradation, which usually resembles physical fission of the vacuolar membrane triggered by osmotic stress rather than nutrient stress. Spermidine is known as a potent inducer of autophagy across many diverse species including yeasts and can prolong lifespan (56, 57). Polyamine flux was also found to remain active under polyamine starvation in *S. cerevisiae* (58). This evidence suggests that our *C. albicans* cells may continue to fight osmotic pressure changes via vacuole fragmentation exerted by excessive polyamines in the absence of exogenous or endogenous polyamines. Therefore, disruption of polyamine homeostasis likely affected the cells more rapidly and severely than gradual polyamine depletion alone. Furthermore, the continuum of vacuolar phenotypes observed, spanning from mild fragmentation and variations in vacuole size and number, may be attributed to heterogeneity within the cell population. This heterogeneity is likely a consequence of altered cell cycle progression due to the disrupted polyamine levels, as polyamines are known to play a crucial role in cell cycle regulation (10, 25).

The differential requirement of polyamines for filamentation and virulence properties is well documented. Herrero et al. (4) reported that the *SPE1* deletion blocked *C. albicans* filamentation on solid Lee’s agar medium with mannitol (pH 6.8); however, these mutants retain virulence in a murine infection model (4). In contrast, Naseem et al. (7) discovered that *spe1* mutants exhibit impaired invasive growth under *in vitro* conditions and are avirulent in a murine model of oropharyngeal candidiasis (7). This difference in virulence properties is not unexpected given the relative abundance of polyamines in host tissues. In the intravenous model, host-derived polyamines likely compensate for biosynthetic defects, whereas limited polyamine availability on the tongue restricts invasive growth. A recent study confirmed that absolute polyamine-deficient mutants are avirulent when both polyamine biosynthesis and transport are disrupted (59), in contrast to an earlier observation reporting an avirulent phenotype with the loss of polyamine biosynthesis alone (4). Lopez et al. (5) reported elevated ODC activity (Spe1) during hyphal growth, although *SPE1* transcript levels remained unchanged during both yeast and hyphal phases of *C. albicans* growth, underscoring the importance of possible post-transcriptional regulation of *SPE1*. In this study, we confirmed that polyamines are absolutely required for *C. albicans* filamentation using polyamine-free medium. Notably, we identified spermine as the primary polyamine driving filamentation, acting synergistically with glucose. The filamentation assays in this study were conducted in SC liquid medium containing 0.2% glucose, a concentration substantially lower than that present in standard SC medium formulations. Glucose titration experiments demonstrated that glucose is required for spermine-induced filamentation (Fig. 4B). However, filamentation was not observed in the absence of polyamine supplementation, indicating that glucose alone is insufficient to induce this phenotype under the conditions tested, suggesting that polyamines act in concert with glucose availability to influence morphogenesis. We selected SC medium because it provides a relatively simple and defined nutritional environment that minimizes the multiple filament-inducing cues present in more complex media, such as RPMI, Spider, and Lee’s medium. This approach allowed us to more directly evaluate the contribution of polyamines to growth and morphogenesis. We further verified that none of the glucose catabolites can replace this glucose requirement, indicating that glucose is likely to act as a signaling molecule rather than a nutritional counterpart.

Most metabolites also serve as signaling molecules for *C. albicans* morphogenesis, including the yeast-to-hyphal conversion (dimorphism), biofilm formation, chlamydospore formation, and immunomodulation both *in vitro* and *in vivo* (2, 60). For example, the amino sugar N-acetylglucosamine (GlcNAc) is catabolized into fructose-6-phosphate enabling the yeast to utilize GlcNAc as the sole carbon source (61–63), while the non-phosphorylated GlcNAc acts as a signal for cAMP-dependent filamentation (reviewed in 64, 65). Interestingly, another study highlighted the importance of GlcNAc signaling via Ngs1 for *C. albicans* persistence in the mammalian gut (66). *GCN4* and the *TOR* pathways represent major responders to amino acid assimilation and sensing in *C. albicans*, which can in turn induce cAMP-*RAS1*-dependent filamentation (67–69). Notably, Schrevens et al. (8) showed that *SPE2* is a major contributor to methionine-induced filamentation, emphasizing the requirement of decarboxylation of SAM for methionine-linked cAMP production that promotes hyphal induction (8). Furthermore, methionine activates the PKA pathway through the production of cAMP in conjunction with the G-protein-coupled receptor (GPCR) Gpr1 (70–72). Similarly, the cAMP pathway is known to be triggered through glucose via Gpr1 in *S. cerevisiae* by separating glucose sensing from glucose catabolism (33, 72). In mammals, biogenic polyamines alone can induce glucose-stimulated insulin gene expression that depends on G-protein signaling pathways (73, 74). These studies suggest that the existence of dual systems is not an uncommon phenomenon in cell signaling. Given our observation of an absolute requirement for glucose in spermine-induced filamentation, here, we propose the presence of two coordinated regulatory mechanisms that integrate polyamine signaling with glucose sensing to induce filamentation in *C. albicans*. To further delineate whether glucose acts through metabolic or sensing pathways, investigating the ability of glucokinase deletion mutants to undergo polyamine-induced filamentation represents an important future direction. Such experiments would help to clarify whether polyamine-induced filamentation depends on glucose metabolism or occurs solely through glucose sensing mechanisms.

Our results indicated that *SPE2* deletion leads to excessive accumulation of putrescine and spermidine. We also noted that adding spermidine, but not spermine, can restore filamentation in polyamine-depleted *Δ/Δspe1*, *Δ/Δspe2*, and *Δ/Δspe1 Δ/Δspe2* mutants. In contrast, in minimal medium (SC liquid), spermine is the only polyamine that was able to restore the filamentation defects. RPMI 1640 is a general filament-inducing medium, and its specific hyphal inducers are not fully known. One notable difference between RPMI 1640 and yeast nitrogen base (a component of SC medium) is their trace element composition. While RPMI is relatively metal-deficient, the yeast nitrogen base (YNB) is formulated with added Fe(II), Zn(II), Cu(II), and Mn(II), providing optimal growth conditions for yeasts (75). Therefore, we presume that the media-dependent polyamine effect could be due to metal ions that facilitate uptake of the exogenously provided polyamines. Most polyamine transporters in yeasts are also responsible for ion uptake, thereby maintaining ion homeostasis (54, 59, 76). A likely explanation is that under the different nutrient environments, the affinity of each polyamine for transporters may differ in the presence of RPMI 1640 and YNB. RPMI 1640, which contains approximately 0.2% glucose and substantially low amino acid levels together with metal deficiency, can rewire metabolic priorities that may hinder the degree of filamentation (77). Therefore, our observation of defective filamentation in RPMI 1640 for all polyamine-deficient mutants may be due to the limited availability of multiple nutritional factors for cell signaling. In the context of polyamine deficiency, impaired metabolic buffering under metal deficiency could further sensitize the cells to low glucose, exacerbating the defects in hyphal elongation despite initial germ tube formation (Fig. 3A). Imported spermidine can be readily converted into spermine, thereby restoring filamentation in RPMI 1640 medium more efficiently than spermine itself. This may be explained by differential uptake of exogenous polyamines under metal deficiency. An additional consideration is that elevated concentrations of spermidine and spermine impaired *C. albicans* growth, raising the possibility that the reduced filamentation observed under these conditions (Fig. 3) may be attributable, at least in part, to growth inhibition or cell cycle arrest. Cell viability was occasionally verified by methylene blue staining prior to our assays. However, whether excess polyamines also influence morphogenesis through specific regulatory pathways independent of growth remains unclear and warrants further investigation. Additionally, one major challenge in culturing polyamine-deficient mutants is inadvertent contamination from exogenous polyamines present in glassware and other media components (78, 79). To minimize this risk, we used acid-washed glassware, filter-sterilized all liquid media, used sterile Falcon tubes for cell culture and filamentation assays, and used Nobel agar for solid media preparation, thereby ensuring the maximal depletion of contaminating polyamines.

In conclusion, the evidence presented here clarifies the fine regulation of *C. albicans* growth and filamentation by polyamines. It is apparent that Spe1 and Spe2 may exert pleiotropic effects on growth and morphogenesis in fungi, extending beyond their canonical roles in a species-dependent manner (10, 80, 81). Given the vital role of spermidine in the hypusination of eIF5A, a critical post-translational modification required for cell growth (48, 82), polyamines may exert broad regulatory control, making it challenging to dissect the specific and independent functions of individual polyamines. Polyamines have been shown to modulate antifungal susceptibility (83–85), providing new avenues for control of fungal infections. Therefore, further studies warrant dissecting the regulatory control mechanisms underlying polyamine function using genetic, biochemical, and system-level approaches.

## MATERIALS AND METHODS

### Culture media and growth conditions

All yeast strains were routinely grown in Yeast Extract-Peptone-Dextrose (YPD; BD Difco™) or on synthetic complete (SC) media-containing agar plates or broth medium, as appropriate, at 30°C unless otherwise stated. SC medium was prepared with BD Difco™ yeast nitrogen base with amino acids (0.67%), glucose (2%), and uridine (0.005%). The filter-sterilized liquid media were used for growth experiments and cell harvesting for filamentation assays, spot assays, and polyamine analysis. The polyamines, spermidine, spermine, and putrescine, were purchased from Goldbio^®^. Supplements for the filamentation assays used include N-acetyl-D-glucosamine, D-(-)-Fructose, D(-)-3-phosphoglyceric acid disodium salt, acetyl-CoA sodium salt, succinyl-CoA sodium salt (purchased through Millipore Sigma™), sodium pyruvate, citric acid monohydrate (purchased through Thermo Fisher Scientific Inc., (Waltham, MA, USA), and sodium acetate (Alfa Aesar, Thermofisher Scientific, Ward Hill, MA, USA). For strain construction, nourseothricin sulfate (NAT) was purchased from Goldbio^®^, and culture media were prepared as described by Nguyen et al. (30). All mutant strains were grown using polyamine-free media and utensils, which involved using glassware that was acid-washed and/or commercially purchased plastic (polypropylene) labware.

### Strain construction

All *C. albicans* strains in this study were derived from the SC5314 wild-type (WT) parent strain. A complete list of the constructed mutants and their genotypes is provided in Table 1. Single gene deletion mutants were constructed using a markerless *HIS-FLP* CRISPR system previously described by Nguyen et al. (30), in which both alleles were deleted by a single transformation step. Following the loop out of the Cas9/gRNA expression cassette, all mutants were heterozygous at the *HIS1* locus. The guide RNA oligos and other oligos were designed using the Benchling (www.benchling.com) cloud-based platform, as described previously (30). A Cas9 expression cassette (from pADH99) and gRNA expression cassette were transformed for integration at the *HIS1* locus of the parent strain (SC5314), along with a 123 bp donor DNA comprised of 50 bp upstream and 50 bp downstream of the coding region for *SPE1* and/or *SPE2*, with an internal ADD-TAG sequence (gRNA target site for generating add-back strains). The oligos used are listed in Table S1. Positive transformants were selected on YPD medium supplemented with 200 μg/mL nourseothricin (NAT), and gene deletions were confirmed by whole-cell PCR and sequencing. The removal of CRISPR components and the NAT marker in the positive transformants was carried out by growing strains in maltose-containing YP medium. NAT-sensitive strains were isolated by plating on YPD containing 25 μg/mL NAT and then patching on YPD + 400 µg/mL NAT plates for verification. The double gene deletion mutants were constructed by simultaneous deletion of both *SPE1* and *SPE2* targets through a single transformation. Add-back strains were generated by integrating WT copies of *SPE1* and *SPE2* at the *Δ/Δspe1* and *Δ/Δspe2* loci, respectively, using a gRNA specific for the ADD-TAG. All add-back strains were verified by whole-cell PCR and gene sequencing.

**TABLE 1:** The yeast strains constructed for this study

Strain	Genotype	Parent	Reference
SC5314	Prototrophic wild-type	Clinical isolate	(86)
*Gene knockout strains*			
RPY300 (*Δ/Δspe1*)	*spe1Δ/spe1Δ HIS1/his1Δ*	SC5314	This study
RPY308 (*Δ/Δspe2*)	*spe2Δ/spe2Δ HIS1/his1Δ*	SC5314	This study
RPY316 (*Δ/Δspe1 Δ/Δspe2*)	*spe1Δ/spe1Δ spe2Δ/spe2Δ HIS1/his1Δ*	SC5314	This study
*Add-back/complemented strains*			
RPY424 (*Δ/Δspe1::SPE1/SPE1*)	*spe1Δ::SPE1/spe1Δ::SPE1 HIS1/his1Δ*	RPY300	This study
RPY392 (*Δ/Δspe2::SPE2/SPE2*)	*spe2Δ::SPE2/spe2Δ::SPE2 HIS1/his1Δ*	RPY308	This study
RPY401 (*Δ/Δspe1::SPE1/SPE1* *Δ/Δspe2::SPE2/SPE2*)	*spe1Δ::SPE1/spe1Δ::SPE1* *spe2Δ::SPE2/spe2Δ::SPE2 HIS1/his1Δ*	RPY316	This study

### Phenotypic assays

For growth kinetics, cells were precultured in polyamine-free SC liquid medium and inoculated at an OD₆₀₀ of 0.1 as the initial cell density. Growth assays were performed using a Thermo Scientific™ Multiskan™ SkyHigh Microplate Spectrophotometer (Thermo Fisher Scientific, Waltham, MA, USA) in 96-well plates, and OD₆₀₀ measurements were recorded every hour for 36 hours at 30°C with intermittent shaking. Experiments were performed in three biological replicates, each with at least six technical replicates.

For spot assays, overnight cultures grown in polyamine-free SC liquid medium were harvested and washed three times with 1X PBS (HyClone, Cytiva, Logan, UT, USA) to remove any residual nutritional molecules. The cell cultures were adjusted to 1 × 10^6^ cells/mL in 1X PBS, and a 1:10 serial dilution series was prepared for each yeast strain in a 96-well plate. Five microliters from each dilution was spotted on freshly prepared polyamine-free SC medium plates supplemented with spermine, spermidine, putrescine, or ornithine at appropriate concentrations.

Filamentation assays were carried out in RPMI medium 1640 (1X) (Gibco™, Thermo Fisher Scientific, Waltham, MA, USA). Cells were grown in SC medium for 24 h at 30°C under aeration, harvested, and washed thrice in 1X PBS buffer (HyClone, pH 7.4) to prepare the inoculum. The cell pellets were washed to remove any cell debris and extracellular excretions. An inoculum of 1 × 10^6^ cells/mL was suspended in pre-warmed RPMI 1640 medium at 37°C and incubated with shaking for 4 h, at which point the cells were fixed to examine for filamentation by light microscopy (EVOS 5000M, Invitrogen Imaging System, Invitrogen™, Thermo Fisher Scientific, Waltham, MA, USA).

### MDY-64 staining

Yeast strains were grown to stationary phase in polyamine-free SC medium without amino acids and briefly washed once with 10 mM HEPES buffer (pH 7.4). The cells were resuspended at 10^6^ cells/ml in 10 mM HEPES buffer containing 5% glucose and stained with yeast vacuole membrane marker MDY-64 (Invitrogen Yeast Vacuole Marker Sampler Kit, Cat. No. Y7531) following the manufacturer’s instructions. The cells were visualized and imaged by fluorescence microscopy (EVOS 5000M, Invitrogen Imaging System, Invitrogen™, Thermo Fisher Scientific, Waltham, MA, USA).

### Polyamine analysis using liquid chromatography–mass spectrometry (LC-MS)

#### Sample preparation

A preculture was prepared by growing yeast cells overnight in 5 mL of polyamine-free SC liquid medium. A 10 µL aliquot from each preculture was used to inoculate 25 mL of SC liquid medium in sterile disposable PETG flasks, and cells were grown for 24 h at 30**°**C with aeration. A culture equivalent to absorbance 20 (i.e., 20 mL from an OD_600nm_ = 1.0 culture) was pelleted at 5,000 rpm for 5 min and washed three times with molecular-grade sterile distilled water. A 5 µL aliquot of the third cell wash was used for cell counting with the Bright-Line hemocytometer. This cell OD adjustment and quantification were necessary for subsequent polyamine calculations. The cell pellet was then flash-frozen and stored at −80°C until analysis. The samples were sent to the Proteomics Core Facility, University of Nebraska–Lincoln, for polyamine analysis through LC-MS.

#### LC-MS analysis

Polyamines and metabolites were extracted from the samples using 400 µL of chilled 5% TCA on a platform shaker at 4°C and 1,300 rpm for 30 min. The samples were then centrifuged at 5,000 rpm for 5 min at 4°C. Supernatants were transferred to a new microfuge tube and neutralized by adding 1 M Tris buffer. The extracted polyamines were derivatized using AccQ-Tag chemistry as described previously (87). The derivatized compounds were then analyzed using the Shimadzu Nexera II UPLC coupled to the Sciex QTRAP 6500+ mass spectrometer equipped with a TurboIonSpray (TIS) electrospray ion source. For LC separation, an ACCQ-TAG ULTRA C18 1.7 um (2.1 × 100 mm, Waters) was used at a flow rate of 0.7 mL/min. The gradient of the mobile phases A (0.1% formic acid in water) and B (0.1% formic acid in 100% acetonitrile) was as follows: 2% B for 0.1 min, to 50% B for 5.9 min, then 90% B for 0.2 min, held at 90% B for 2.3 min, and then back to 2% B for 0.1 min for a total runtime of 12 min. The QTRAP 6500+ mass spectrometer was tuned and calibrated according to the manufacturer’s recommendations. The instrument was set up to acquire in positive mode. Analyst software (version 1.7.2) was used to control sample acquisition and data analysis. The metabolites ornithine, putrescine, spermidine, and spermine were detected using MRM (multiple reaction monitoring) transitions that were previously optimized using standards. For quantification, an external standard curve was prepared using a series of standard samples containing different concentrations of metabolites and a fixed concentration of the internal standard, 50 mM 1,3-diaminopropane. Concentration of each polyamine was calculated as µg per 10^8^ cells. Intracellular pool results were then analyzed using GraphPad Prism 10.0.2 to generate stacked bar graphs.

## Data Availability

All data supporting the conclusions of this study are contained within the manuscript, figures, and supplemental materials. Additional raw data used for analysis and figures will be made available upon reasonable request.

## SUPPLEMENTAL MATERIAL

Supplemental figures spectrum.00833-26-s0001.pdf Fig. S1 to S4.

Supplemental legends spectrum.00833-26-s0002.docx Legends for Fig. S1 to S4.

Table S1 spectrum.00833-26-s0003.pdf Primers used for this study.

ASM does not own the copyrights to Supplemental Material that may be linked to, or accessed through, an article. The authors have granted ASM a non-exclusive, world-wide license to publish the Supplemental Material files. Please contact the corresponding author directly for reuse.
