# Supplementary material for: Distinct polyamine effects on Candida albicans morphogenesis identify spermidine as a key regulator
Source: Microbiol Spectr. 2026 Sep 23;14(11):e00833-26. doi: 10.1128/spectrum.00833-26 (PMC13729115; doi:10.1128/spectrum.00833-26)
Supplement: Supplementary file 1 [file spectrum.00833-26-s0001.pdf]

Fig. S1 A

```
CLUSTAL O(1.2.4) multiple sequence alignment


CaSpe1p         MKHTIIENEPEIKLQNDLNHH----IEIS------PNTKTTNGTTTTTTTLNV--------      43
ScSpe1p         --------MSSTQVGNALSSSTTTLVDLSNSTVTQKKQYYKDGETLHNLLLELKNNQDLE      52
SpSpe1p         --------MPAI--------------------MEKNMLVSESLRTTELLGHVKPIDSVV      31
                                                    :   .:.        .:

CaSpe1p         --NKTLKAIDLIETSIKNHISTID--HENCLPNDEDSFFVCDLGEIINSVNQWQQILPMV      99
ScSpe1p         -LLPHEQAHPKIFQALKARIGRIN--NETCDPGEENSFFICDLGEVKRLFNNWVKELPRI      109
SpSpe1p         TWSSPGSSRQAIGEAFKNTIEEIERAAVRGEPADSDAFFVADLNGVYRQLLRWHAKLPRV      91
                      .:   *  ::*  *  *:       * :.::**:.**. : . . .*   ** :

CaSpe1p         QPYYAVKCNSNPQILTTLSELGVNFDCASKNEIDLVLSLGIHQAHERIIYANPCKTNSFI      159
ScSpe1p         KPFYAVKCNPDTKVLSLLAELGVNFDCASKVEIDRVLSMNIS--PDRIVYANPCKVASFI      167
SpSpe1p         QPFYAVKCNPDPKVLALLNKFGTGFDCASKGELEQILGLGVS--PDRIVYANPCKAITYV      149
                :*:****** : ::*: * ::*..****** *:: :*.:.:    :**:******. :::

CaSpe1p         RHAADENVNLTTVDNVHELYKLAKFHPHCKILIRLITDDSTAQCQLSTKFGCDLNTAIGE      219
ScSpe1p         RYAASKNVMKSTFDNVEELHKIKKFHPESQLLLRIATDDSTAQCRLSTKYGCEMEN-VDV      226
SpSpe1p         RYAASKGINLMTFDNADELYKVKQHHPNSRLLLRISTDDSNSLCRLSLKFGASLDD-TGK      208
                *:**.:.:   *.**..**:*: :.**..::*:*: ****.: *:** *:*..::   .

CaSpe1p         ILPKAKELGLQVHGVAFHVGSGAKDFSSIYQAIKDSRILFDEMLSM--GFTPKLLDIGGG      277
ScSpe1p         LLKAIKELGLNLAGVSFHVGSGASDFTSLYKAVRDARTVFDKAANEYGLPPLKILDVGGG      286
SpSpe1p         LLDIAKSLELNVVGVSFHVGSGSYDPSAFLDAIQRSRQVFDQGLER--GFNFDLLDIGGG      266
                :*   *.* *:: **:******: * ::: .*:: :* :**:  .       .:**:***

CaSpe1p         FERETFPQSSQMVKFALEKFFPIEFSQLNEIKFIAEPGRFMVANAFTLITHIIARRDLPT      337
ScSpe1p         FQFESFKESTAVLRLALEEFFPVGC----GVDIIAEPGRYFVATAFTLASHVIAKRKLSE      342
SpSpe1p         FMNDSFDGVADLIRSALDTYFDP------SIRVISEPGRFFVSSSFTLAVNVIAKRKLDD      320
                *  ::*   : ::: **: :*         : .*:****::*:.:***  ::**:*.*

CaSpe1p         GGNNNNNDMTPSAMLYINDGVYGNLNCILFDHQTPKVYVLTNENQLFYKQEMMRSLSVNN      397
ScSpe1p         N---------E-AMIYTNDGVYGNMNCILFDHQEPHPRTLYHNLEFHYDDFESTT-AVLD      391
SpSpe1p         E---------EKVMYYVNDGVYGSLNCILFDHQHPVARVLKCGSRFVYNDLVGTG-----      366
                            .* * ******.:******** *   .*    .: *.:

CaSpe1p         NNNNNNKTDGFKFSIWGPTCDGLDCVSSLAKLSKNVQVGDWLFFENVGAYTSCASTKFNG      457
ScSpe1p         SINKTRSEYPYKVSIWGPTCDGLDCIAKEYYMKHDVIVGDWFYFPALGAYTSSAATQFNG      451
SpSpe1p         ---------QHRCFIWGPTCDSLDVIANDAHLPYELNVGDWIYFEDAGAYTVAAASCFNG      417
                          .:  *******.** ::.   :  :: ****::*   **** .*:: ***

CaSpe1p         LSSGETKTLYVNSNEE-   473
ScSpe1p         FEQTAD-IVYIDSELD-   466
SpSpe1p         FKTS-R-IVYLDTDILD   432
                :.      :*::::
```

```
Percent Identity  Matrix - created by Clustal2.1


  1: CaSpe1p     100.00    46.64    43.06
  2: ScSpe1p      46.64   100.00    45.67
  3: SpSpe1p      43.06    45.67   100.00
```

Fig. S1 B

```
CLUSTAL O(1.2.4) multiple sequence alignment


CaSpe2p      -----MVAPALIYSNHELSTAIDSTHAFEGPEKLLEIWFYESKEL-------SPINLRDI     48
ScSpe2p      MTVTIKELTNHNYIDHELSATLDSTDAFEGPEKLLEIWFFPHKKSIT-----TEKTLRNI     55
SpSpe2p      -------MSPTLVVDQENS-EEFNTSSFEGPEKLLELWFSAPIKTNLSAGEKANLGLKAV     52
                           ::* *    .* :*********:**    :        :   *: :

CaSpe2p      IFDTWIEILNLVHCEVLSKVSSNLCDAFLLSESSLFVFPHKIILKTCGTTTTLACLDLLF     108
ScSpe2p      GMDRWIEILKLVKCEVLSMKKTKELDAFLLSESSLFVFDHKLTMKTCGTTTTLFCLEKLF     115
SpSpe2p      SRNDWDDMLAQAQCKVLSVVNSEEIDAYLLSESSMFVFAHKIILKTCGTTTLLASLPRLL     112
               : * ::*  .:*:***  .::  **:******:*** **: :******* * .*  *:

CaSpe2p      ETVNKELLQNEGLKATFQSKNIYQIFYSRRSFMFPDRQIHVHGNWQEEVKLLNQYFNNGK     168
ScSpe2p      QIVEQELSWAFRTT-QGGKYKPFKVFYSRRCFLFPCKQAAIHQNWADEVDYLNKFFDNGK     174
SpSpe2p      EIAS-----------SVGFDRPLRIFYSRKNFLYPERQLAPHTSWEEEVRYLQLFFPSGC     161
             : ..                .  ::****: *::* :*   * .* :**  *: :* .*

CaSpe2p      SYIVGNN---TNWHLYVGGNGKTKNPVASTTTTTTTPVVNDCTLEIIMTQLSLEASQQFY     225
ScSpe2p      SYSVGRNDKSNHWNLYVTETDRSTPKG------KEYIEDDDETFEVLMTELDPECASKFV     228
SpSpe2p      SYVVGPTNK-NHWHLFSDLADDYSL-------LEDSLDPEDETLEVLMTDMSPERSLQFY     213
             ** ** .   .:*:*:    .  .               :* *:*::**::. * : :*

CaSpe2p      TTRKPG-DT-------AIDSNHDLGHDLGQEILKQTGLNELFKFKKQPTMPGLSSSPIKE     277
ScSpe2p      CGPEASTTA-------LVEPNEDKGHNLGYQMTKNTRLDEIYVNSAQ-------DSDLSF     274
SpSpe2p      APSLDVVRSARGDDYVREKNNLSGGHILGSYVADESGVRDLC-----------STSDKKA     262
                     :         . * . ** **  : .:: : ::              *  .

CaSpe2p      IHDGFAFTPCGFSSNSIN-ESNYYTIHVTPEPGWSYASFETNMIG--------DYKAIVD     328
ScSpe2p      HHDAFAFTPCGYSSNMILAEKYYYTLHVTPEKGWSYASFESNIPVFDISQGKQDNLDVLL     334
SpSpe2p      VLDAFQFEPIGFSSNMIY-KDRYATIHVTPQEHCSYASFETNVSQFQFGRSISE---TIE     318
               *.* * * *:*** *  :. * *:****:   ******:*:          :    :

CaSpe2p      KCINVFQPGQFMVTFLTNTDME-------KFECCLDDYKVNHREELDVEDYKLYFVEFVR     381
ScSpe2p      HILNVFQPREFSMTFFTKNYQNQSFQKLLSINESLPDYIKLDKIVYDLDDYHLFYMKLQK     394
SpSpe2p      KTVKTFGANKFCLTLFQAKGASQEKH-FSAKLKSFSSYKREEFIVYDFPGYDLIFASFTA     377
             : ::.*   :* :*::  .  .           .: .*   .    *. .*.* : .:

CaSpe2p      Q- 382
ScSpe2p      KI 396
SpSpe2p      V- 378
```

```
Percent Identity  Matrix - created by Clustal2.1


  1: CaSpe2p     100.00   45.11   38.33
  2: ScSpe2p      45.11  100.00   36.81
  3: SpSpe2p      38.33   36.81  100.00
```

Fig. S2

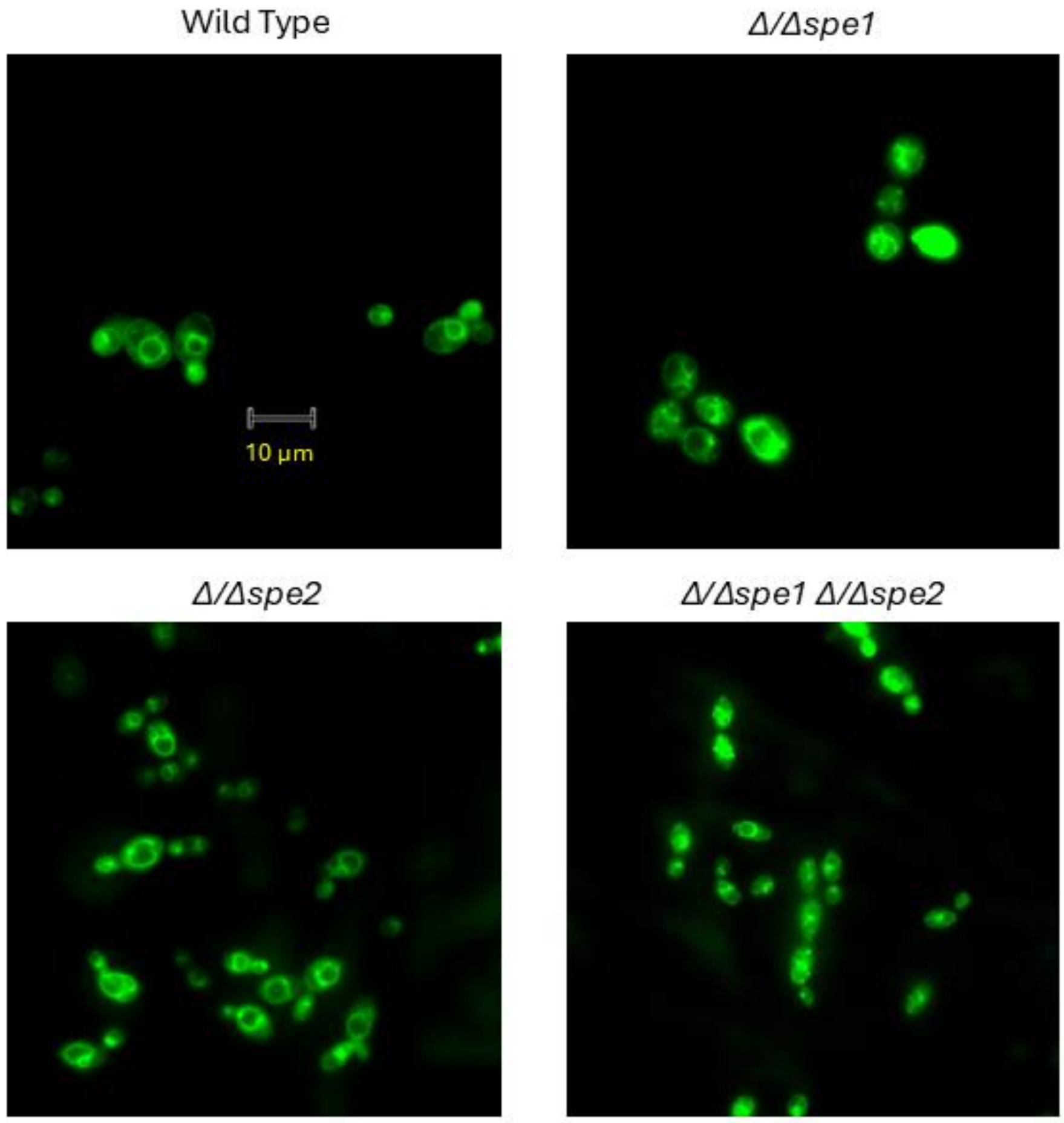

Fig. S3

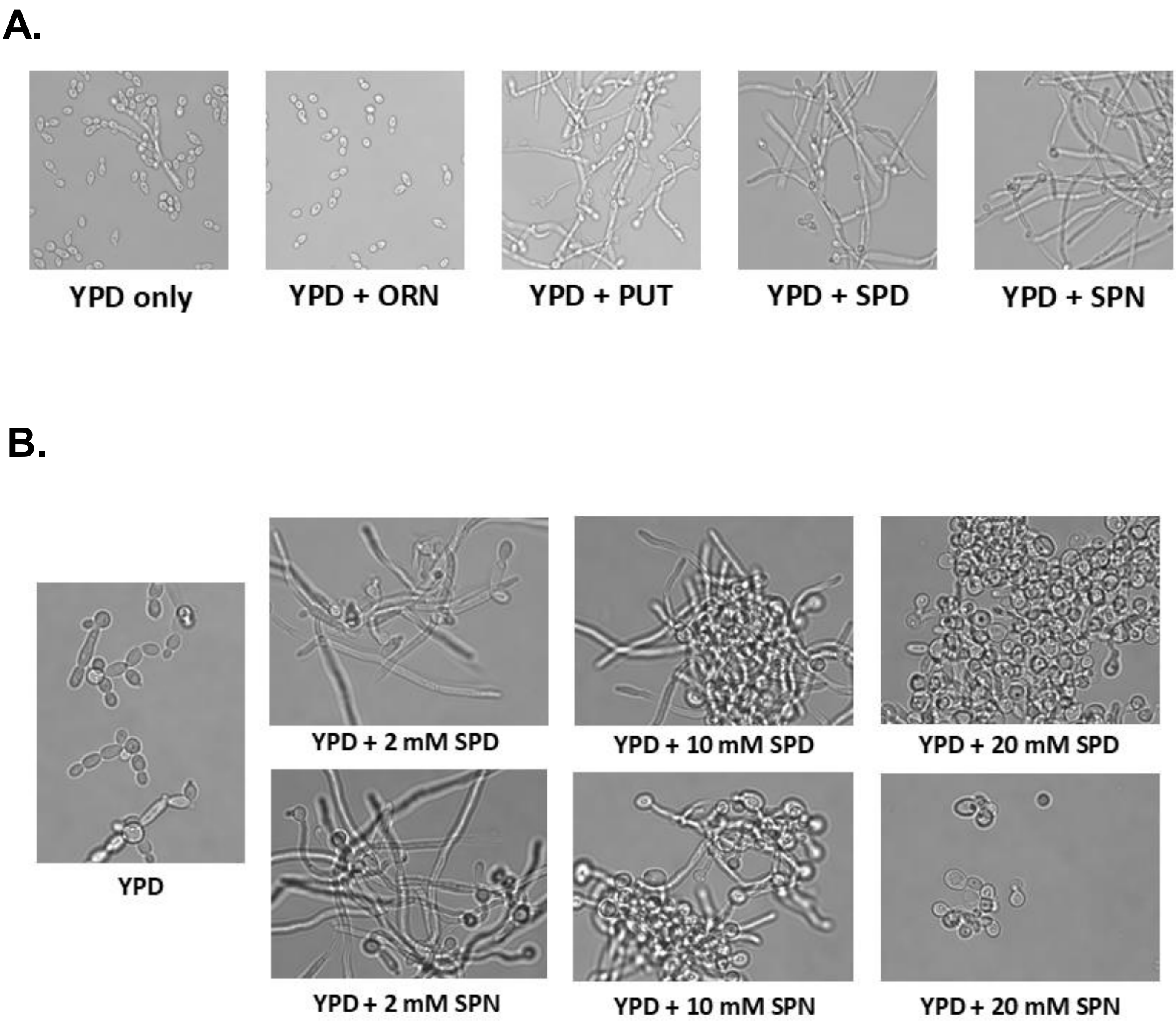
A.
YPD only
YPD + ORN
YPD + PUT
YPD + SPD
YPD + SPN
B.
YPD
YPD + 2 mM SPD
YPD + 10 mM SPD
YPD + 20 mM SPD
YPD + 2 mM SPN
YPD + 10 mM SPN
YPD + 20 mM SPN

Fig. S4

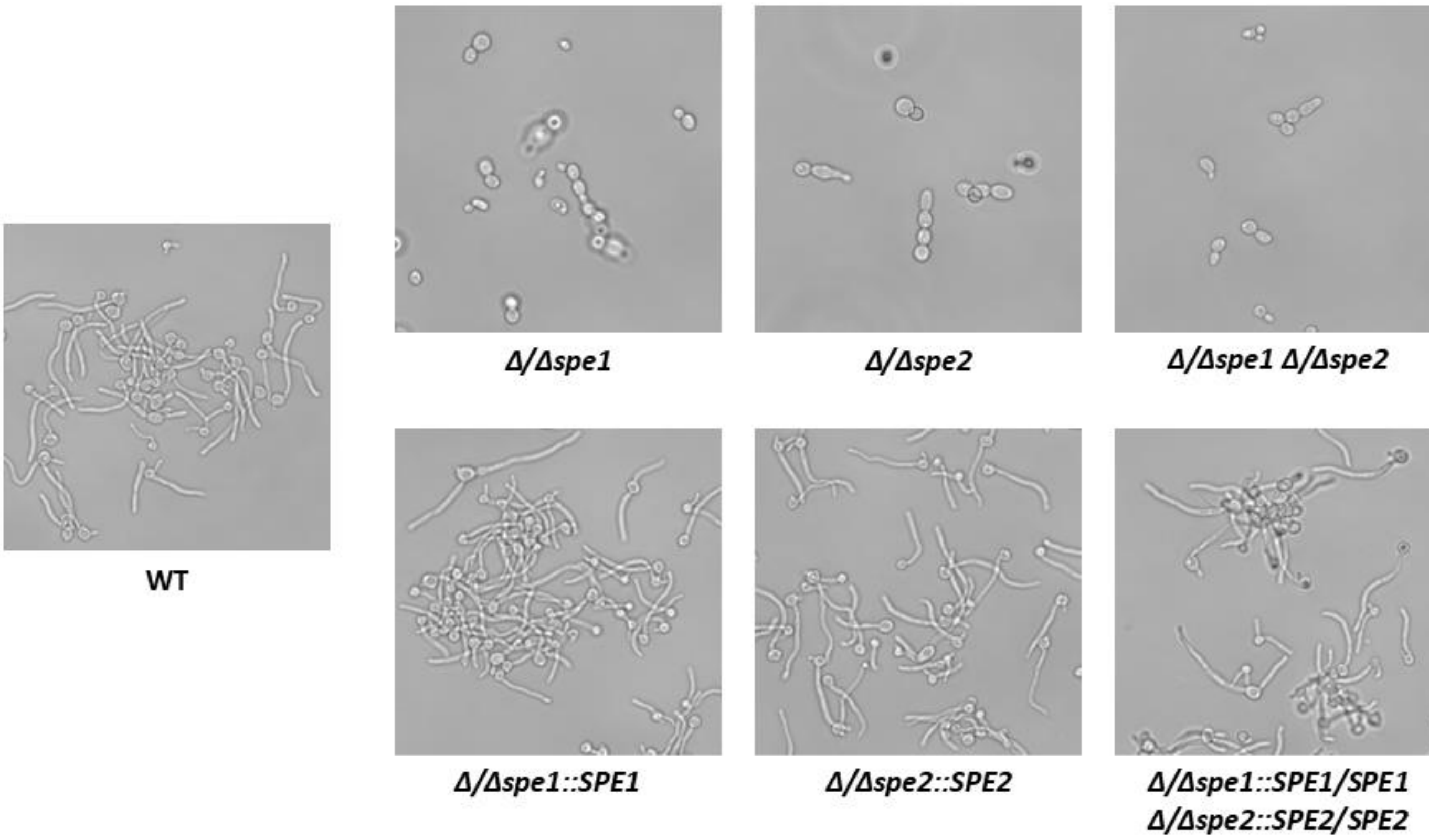
WT
Δ/Δspe1
Δ/Δspe2
Δ/Δspe1 Δ/Δspe2
Δ/Δspe1::SPE1
Δ/Δspe2::SPE2
Δ/Δspe1::SPE1/SPE1
Δ/Δspe2::SPE2/SPE2
