# Supplementary material for: Distinct polyamine effects on Candida albicans morphogenesis identify spermidine as a key regulator
Source: Microbiol Spectr. 2026 Sep 23;14(11):e00833-26. doi: 10.1128/spectrum.00833-26 (PMC13729115; doi:10.1128/spectrum.00833-26)
Supplement: Supplementary file 2 [file spectrum.00833-26-s0003.pdf]

Table S1: Primers used for this study

Primer Name	Primer Sequence	Primer Description
Gene Deletion		
RPO117	CGTAAACTATTTTTAATTTGAGACGTGATTTACCAACGGGGTTTTAGAGCTAGAAATAGC	CRISPR gRNA for *SPE1* deletion
RPO118	AAAGTGAAAAAGATCAGAGAAAGAGAGTATAAGCTATCCTTTTCCAAGTTCGAGACGAGTGCTCGACATGAGG	5' flank + ADD-TAG for *SPE1* KO
RPO119	TTCATATTAATTGAATACATAAATAAGTAAACACATATACCTATAGATTCCCTCATGTCGAGCACTCGTCTCG	3' flank + ADD-TAG for *SPE1* KO
RPO127	CGTAAACTATTTTTAATTTGGAATTACTAGAAAATCCACAGTTTTAGAGCTAGAAATAGC	CRISPR gRNA for *SPE2* deletion
RPO128	CAACAACAACAGCAAAAACAACTAGGATACAACACCAAAGTTACCTGACACGAGACGAGTGCTCGACATGAGG	5' flank + ADD-TAG for *SPE2* KO
RPO129	CACTAAGCAATCTAAAAACAAAATATTAAAATAACACCTTATAGGAGCACCCTCATGTCGAGCACTCGTCTCG	3' flank + ADD-TAG for *SPE2* KO
Gene Add-back/Complementation		
TNO16	CGTAAACTATTTTTAATTTGCGAGACGAGTGCTCGACATGGTTTTAGAGCTAGAAATAGC	CRISPR gRNA for ADD-TAG
RPO145	CCTGCCCCAAATTTACTTGA	*SPE1* FW dDNA for ADD-BACK
RPO146	TCGTCAACCAACCAACAAG	*SPE1* REV dDNA for ADD-BACK
RPO147	TCCCAATTTCCAATCAAGAGTG	*SPE2* FW dDNA for ADD-BACK
RPO148	TTTGCACGACACACAATCTAC	*SPE2* REV dDNA for ADD-BACK

Abbreviations: gRNA = guide RNA; dDNA = donor DNA; red = ADD-TAG sequence.
