# Supplementary material for: Distinct polyamine effects on Candida albicans morphogenesis identify spermidine as a key regulator
Source: Microbiol Spectr. 2026 Sep 23;14(11):e00833-26. doi: 10.1128/spectrum.00833-26 (PMC13729115; doi:10.1128/spectrum.00833-26)
Supplement: Supplementary file 3 [file spectrum.00833-26-s0002.docx]

**Fig. S1: Alignment of *SPE1* and *SPE2* homologs in *C. albicans*, *S. cerevisiae* and *S. pombe*.** Multiple sequence alignment of (**A**) Spe1 and (**B**) Spe2 protein sequences and percent identity matrices were obtained with Clustal Omega ([www.ebi.ac.uk](http://www.ebi.ac.uk)) using default parameters for the amino acid sequences obtained for *S. cerevisiae* (*Saccharomyces* Genome Database: *SPE1* / *YKL184W* and *SPE2* / *YOL052C*), *S. pombe* (PomBase: *spe1* / *SPAC144.04c* and *spe2* / *SPBP4H10.05c*) and *C. albicans* (*Candida* Genome Database: *C1_00740C_A*|*SPE1* and *SPE2*/*C5_00700C*). Ca = *C. albicans*; Sc = *S. cerevisiae*; Sp = *S. pombe.* Red = hydrophobic residue; blue = acidic residue; green = hydroxyl + sulfhydryl + amine + Glycine; magenta = basic residue-Histidine. In the bottom row, the alignment results are represented as follows: the asterisk (*) indicates a single, fully conserved residue. A colon (:) indicates conservation between groups with strongly similar properties, and a period (.) indicates conservation between groups with weakly similar properties.

**Fig S2: Vacuole morphology of polyamine-deficient mutants.** Representative fluorescent images of the indicated strains, WT, *Δ/Δspe1, Δ/Δspe2*, and *Δ/Δspe1 Δ/Δspe2,* following vacuolar staining with MDY-64 (Invitrogen™). Yeast cells grown to stationary phase in minimal medium were stained with MDY-64 and observed by fluorescence microscopy. For the WT strain, the staining pattern reveals well-defined spherical or ellipsoid vacuoles, one per non-budding cell, while the mutants exhibit altered vacuole staining patterns, such as enlarged, fragmented or multiple or irregular shapes, indicated by disrupted, wrinkled or discontinued fluorescent rings. Scale bar indicates 10 µm.

**Fig. S3**: **Dose-dependent filamentation response to polyamines in rich medium (YPD) at 37 ⁰C. A.** In rich medium (YPD), polyamines spermine (SPN), spermidine (SPD) and putrescine (PUT) strongly induce *C. albicans* filamentation, while the precursor ornithine (ORN) fails to trigger hyphal growth when cells are grown at 37 °C for 4 hrs. **B.** *C. albicans* can tolerate higher levels of spermidine and spermine in rich medium, which induce filamentation up to 10 mM concentrations. At very high concentrations, filamentation is retarded likely due to the toxic effects of excess polyamines.

**Fig S4.** **Restoration of filamentation phenotypes in add-back strains of *C. albicans* in comparison with the WT and gene-deleted mutant strains.** Representative bright-field images of cell morphology of the indicated strains following hyphal induction in RPMI 1640 medium at 37°C for 4 hrs.
